# Effects of short‐term exposure to moderate amounts of alcohol on brain volume

**DOI:** 10.1002/npr2.12500

**Published:** 2024-12-12

**Authors:** Sakiko Tsugawa, Fumihiko Ueno, Mutsuki Sakuma, Hideaki Tani, Ryo Ochi, Ariel Graff‐Guerrero, Yoshihiro Noda, Hiroyuki Uchida, Masaru Mimura, Shunji Oshima, Sachio Matsushita, Shinichiro Nakajima

**Affiliations:** ^1^ Department of Neuropsychiatry Keio University School of Medicine Tokyo Japan; ^2^ Multimodal Imaging Group, Research Imaging Centre Centre for Addiction and Mental Health (CAMH) Toronto Ontario Canada; ^3^ Sustainable Technology Laboratories Asahi Quality and Innovations, Ltd Moriya‐Shi Ibaraki Japan; ^4^ National Hospital Organization Kurihama Medical and Addiction Center Yokosuka Kanagawa Japan

**Keywords:** neuroimaging: structural

## Abstract

**Aim:**

Although numerous studies have reported that chronic alcohol consumption causes brain volume reduction and cerebrospinal fluid volume increase, few studies have examined the acute effects of alcohol on brain structure. This study aims to investigate the short‐term brain volume changes following alcohol administration.

**Methods:**

Moderate doses of alcohol were administered intravenously to 18 healthy volunteers for a total of 90 min to achieve a blood alcohol concentration of 0.5 mg/mL. An alcohol clamp method combined with physiologically based pharmacokinetic modeling was used to achieve fine control over blood alcohol concentration. T1 images with 3T MRI were scanned at three time points: baseline, 0 min, and 90 min after the end of alcohol administration. Cortical, subcortical, and ventricular volumes were computed after segmentation with FreeSurfer. Repeated measures analysis of variance was used to evaluate longitudinal changes in brain volume at 96 regions.

**Results:**

Acute alcohol administration increased bilateral lateral ventricular volumes, which lasted until 90 min after the end of alcohol injection. On the other hand, the volumes of total gray matter, left precentral cortex, left caudal middle frontal cortex, and left superior frontal cortex decreased after alcohol administration, but these changes disappeared 90 min after the end of alcohol administration.

**Conclusion:**

Acute injection of moderate doses of alcohol may enlarge ventricle volumes and reduce gray matter volumes. The transient volume changes caused by acute administration of alcohol may be related to changes in CSF flow and water content of brain tissue, which warrants further study.

## Abstract 2

This study reveals short‐term brain changes from moderate alcohol doses in healthy volunteers. MRI scans show an increase in lateral ventricular volumes and a decrease in specific gray matter regions. These changes suggest acute alterations in CSF dynamics and brain water content, highlighting the need for further research into the underlying biological mechanisms.

## Graphical abstract

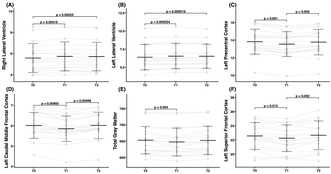

## 1 INTRODUCTION

Alcohol use disorder (AUD) is a worldwide concern responsible for 3 million deaths each year on a global scale and for 5.1% of the global burden of disease.^1^ Neurotoxicity of heavy chronic alcohol consumption can result in global gray matter (GM) volume reductions which may be associated with cognitive impairment in AUD.^2^, ^3^, ^4^, ^5^ A recent meta‐analysis reported that patients with AUD had GM volume loss in the regions related to specific cognitive, emotional, somatosensory, and motor functions such as the insula, cingulate cortex, paracentral lobes, post‐ and precentral cortex, and superior frontal cortex.^6^ Also, greater scores on drink index measuring quantity, frequency, intoxication, and maximum number of drinks were associated with decreased thickness in regions contributing to flexible behavioral adaptation and salience detection processes to facilitate successful inhibitory/cognitive control.^7^ On the other hand, the cerebrospinal fluid (CSF) volume in the brain was increased in patients with AUD compared with healthy controls (HC).^8^

Although most prior studies have examined the long‐term effects of heavy alcohol consumption, brain structural change following acute alcohol intake has scarcely been investigated. To the best of our knowledge, only one study investigated transient effects on brain volumes after drinking in healthy subjects. Furtmann et al.^9^ found that cerebral water content and volumes of white and gray matter in healthy subjects did not differ between before and after acute heavy alcohol consumption reaching a breath alcohol concentration (BrAC) of 1.0 mg/L. Besides an enlargement of the CSF volume by chronic alcohol consumption was well known, there is no study which reported CSF volume alteration in the human brain by acute alcohol injection thus far. Further studies are needed to examine acute effects of alcohol on brain structure. To assess the acute effects of alcohol, oral administration has a limitation of three‐ to four‐fold variability of the time course in blood alcohol concentration (BAC) that was mainly due to individual differences in pharmacokinetics. An alcohol clamp method combined with physiologically based pharmacokinetic (PBPK) modeling is more suitable since this can achieve fine control over the rate, magnitude, and duration of exposure to alcohol within a range of 0.05 mg/mL.^10^, ^11^

Therefore, the objective of this study was to investigate the effect of acute and moderate alcohol injection on the volume of all brain regions including cortex, subcortex, and CSF, using the alcohol clamp technique. A secondary objective of this study was to explore the relationship between the changes in brain volume caused by alcohol administration and the clinical response in healthy subjects.

## 2 METHOD

### 2.1 Subject selection and study design

**FIGURE 1:**
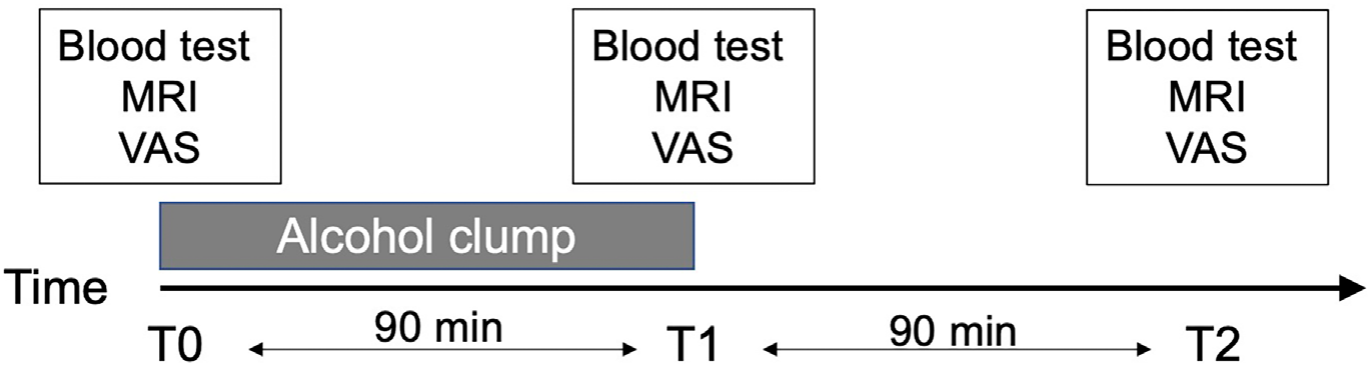
Timeline of experimental session. VAS, visual analogue scales.

This study was approved by the review board of National Hospital Organization Kurihama Medical and Addiction Center, Yokosuka, Japan (Approval ID: 320‐2). The study procedures were performed in accordance with the principles of the Declaration of Helsinki. After the explanation of the purpose and details of this study, written informed consent was obtained from all subjects. The details of inclusion criteria and clinical assessments were described in our previous studies.^12^ We prospectively scanned 18 subjects (11 females) aged between 20 and 29. Inclusion criteria were absence of physical or mental illnesses, right handedness, non‐smoker, drinking alcohol only once or twice a month, and carrier of the ALDH2*1/*2 genotype which is associated with high sensitivity to alcohol.^13^ Individuals possessing the ALDH2*1/*1 genotype were excluded from this study due to their substantially low BAAC, which hinders the effective observation of alcohol sensitivity.^14^ Correspondingly, those with the ALDH2*2/*2 genotype were also omitted due to their pronounced potential for extreme sensitivity or intolerance to alcohol. Exclusion criteria were conditions impeding an MRI examination, taking any medications, a history of drug or food allergies, and a history of drug dependence or abuse. Subjects underwent an IV alcohol infusion using the clamp method to achieve a target BAC of 0.5 mg/mL according to BrAC. Alcohol was injected to reach the target BrAC of 0.25 mg/L in 15 min and maintain it at the target level for a total infusion duration of 90 min. Breath samples were obtained every 5 min throughout the experiment. MRI data and clinical symptoms measured by visual analogue scales (VASs) were acquired at baseline (T0), 90 min after alcohol administration was started (T1) and 90 min after alcohol administration was finished (i.e., 180 min from baseline). Timeline of this experimental session is presented in Figure 1.

### 2.2 Alcohol clamp technique

This study used the alcohol clamp technique developed at the Indiana Alcohol Research Center to maintain the target BrAC in subjects of this study.^11^, ^15^ In order to minimize experimental variance in BrAC levels, 6% alcohol in Ringer's lactate solution was administered intravenously according to a PBPK model. The infusion profile of alcohol solution was precomputed to follow the desired time course of BrAC, i.e., linear ascension to a target BrAC of 0.25 mg/L at 15 min, followed by a steady BrAC maintained.^16^ BrAC was measured using an Alco‐Sensor® IV (Intoximeter, Inc., St. Louis, MO, USA). The infusion rate was adjusted to achieve a target BAC of 0.5 mg/mL according to measurements of BrAC obtained frequently during the session.

### 2.3 Clinical assessment

Participants' clinical responses to alcohol administration were assessed using the VAS at T0, T1, and T2^17^ to confirm the effects of the administered alcohol, which was at an appropriate concentration to elicit some physiological impact, and to investigate its associations with brain volume changes. Participants were instructed to rate the 30 items for clinical responses to alcohol using a 10 cm scale with 0 cm being “as usual” and 10 cm being “very much applicable.” These 30 items, partially derived from the Bodily Sensation Scale^18^ and the Biphasic Alcohol Effects Scale,^19^ were categorized into four groups: stimulant, sedative, physical, and craving, as shown in Table S1. Scores for each category were calculated as the mean length.

### 2.4 MRI data acquisition and preprocessing

A high‐resolution T1‐weighted anatomical image was acquired for each participant at Kurihama Medical and Addiction Center in a 3T GE Discovery MR750 scanner (GE Healthcare, Wauwatosa, WI, USA) using a three‐dimensional fast‐field echo sequence (Axial MRI 3D Brain volume [BRAVO], echo time = 2.8 ms, repetition time = 6.4 ms, inversion time = 650 ms, flip angle = 8°, field of view = 230 mm, 256 × 256 matrix, slice thickness = 0.9 mm). T1‐weighted imaging data were processed with FreeSurfer software version 6.0 (http://surfer.nmr.mgh.harvard.edu). T1 images and segmented labels were visually checked following quality control protocol.^20^ We obtained volumetric measurements for the total GM, total white matter, and 94 distinct regions using FreeSurfer's default atlases; specifically, the Desikan‐Killiany atlas for cortical segmentation and the Aseg atlas for subcortical segmentation.^21^

### 2.5 Statistical analysis

Statistical analyses were carried out using R (version 4.0.2). After testing for data distribution with a Shapiro–Wilk test, repeated measure analysis of variance (ANOVA) was used to examine differences in brain volume among T0, T1, and T2. False Discovery Rate (FDR) was used to correct *p* values for multiple comparisons of 96 structural values and a threshold of *p* < 0.05 was used. Post‐hoc pairwise *t* tests were performed for the ROIs which showed significant differences in the repeated measure ANOVA. Repeated measure analysis of covariance (ANCOVA) was also conducted controlling for age and sex. Multiple regression controls for age and sex were used to predict VAS score changes from baseline volumes or volumetric changes. Additionally, we examined correlation between volumetric changes in the left or right lateral ventricle and total GM. Continuous variables are presented as mean ± standard deviation.

## 3 RESULTS

### 3.1 Characteristics of the participants

Baseline characteristics of all subjects are shown in Table 1. The subjects achieved BrAC of 0.246 ± 0.013 mg/L (range, 0.21–0.27) and BAC of 0.481 ± 0.040 mg/mL (range, 0.40–0.54) immediately after completion of alcohol administration. At 90 min after completion of alcohol administration, the BrAC and BAC were 0.122 ± 0.021 mg/L (range, 0.09–0.16) and 0.309 ± 0.043 mg/mL (range, 0.22–0.36), respectively. Table S1 displays changes in VAS scores, with sedative and physical scores increasing over a period of 180 min.

**TABLE 1:** Demographics and clinical characteristics of the participants.

	Total (*N* = 18)
Age, mean (SD), years	24.8 (3.0)
Sex, female, *n* (%)	11 (61.1)
Height, mean (SD), cm	165.5 (7.5)
Weight, mean (SD), kg	59.4 (8.3)
Total intracranial volume, mean (SD), cm^3^	1507.0 (98.9)

### 3.2 Brain volume alteration

**FIGURE 2:**
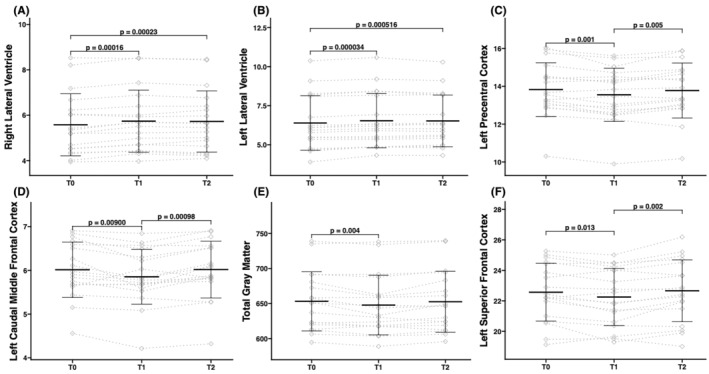
Changes in the brain structure. Brain volumes in (A) the right lateral ventricle, (B) the left lateral ventricle, (C) the left precentral cortex, (D) the left caudal middle frontal cortex, (E) total gray matter, and (F) the left superior frontal cortex were compared across three time points: T0, T1, and T2. Mean brain volumes are represented by horizontal bars. Error bars indicate the standard deviation, and dotted lines depict the individual time courses of brain volumes.

Significant differences were found in the volumes of total GM, left precentral cortex, left caudal middle frontal cortex, left superior frontal cortex, and bilateral lateral ventricles (Figure 2 and Table 2). These significant differences remained after adjusting for age and gender using ANCOVA; however, for the total GM volume, the differences diminished to a trend level (*F*(2,30) = 6.5, *p*_FDR_ = 0.069). Post‐hoc analyses revealed that bilateral lateral ventricle volumes increased at T1 and T2 compared with T0 while there was no difference between T1 and T2. In the left precentral cortex, left caudal middle frontal cortex, and left superior frontal cortex, volumes decreased at T1 from T0 and subsequently increased at T2 compared to T1. No significant differences were observed between T0 and T2. Meanwhile, total GM volumes decreased at T1 from T0, with no significant differences observed between T0 and T2, or between T1 and T2. There was no significant association between baseline structure or structural changes and VAS score changes at T1 or T2 from T0. Also, no significant correlation was observed between changes in volumes of the left or right lateral ventricle and total GM.

**TABLE 2:** Changes in the brain volumes through 180 min.

Time	T0	T1	T2	ANOVA	
				*F*‐value	*p*‐value
Right lateral ventricle	5.58 (1.37)	5.74 (1.37)	5.72 (1.35)	*F*(2,34) = 17.2	<0.0001
Left lateral ventricle	6.39 (1.74)	6.54 (1.74)	6.53 (1.65)	*F*(2,34) = 15.5	<0.0001
Left precentral cortex	13.82 (1.42)	13.55 (1.41)	13.77 (1.45)	*F*(2,34) = 9.8	0.0004
Left caudal middle frontal cortex	6.01 (0.63)	5.85 (0.63)	6.02 (0.65)	*F*(2,34) = 9.0	0.0007
Total gray matter	653.19 (42.20)	647.84 (42.51)	652.57 (43.39)	*F*(2,34) = 7.2	0.003
Left superior frontal cortex	22.57 (1.90)	22.25 (1.88)	22.66 (2.03)	*F*(2,34) = 7.1	0.003

## 4 DISCUSSION

We examined the acute effect of moderate‐dose alcohol injection on brain structure in healthy subjects. Our findings revealed that left and right lateral ventricular volumes increased by alcohol injection and the enlarged volume remained until 90 min after completion of alcohol injection with small effect sizes. On the other hand, volumes in the total GM, left precentral cortex, left caudal middle frontal cortex, and left superior frontal cortex were decreased after alcohol administration with small effect sizes and these changes disappeared 90 min after alcohol administration ended. There was no association between the brain structural alteration and clinical changes by acute alcohol administration.

The strengths of our paper are as follows. First, this is the first human MRI study to comprehensively investigate the structural change including ventricles in acute response to moderate‐dose alcohol. Second, using alcohol clamp technique, BrAC was strictly maintained approximately 0.25 mg/L for any individual. Third, we included only the participants who were genetically sensitive to alcohol (i.e., ALDH2*1/*2 carriers), which is ideal for assessing the acute effects of alcohol. Finally, we scanned the MRI at three time points so that brain structures were evaluated not only in the sober state but also in the recovering state.

### 4.1 Increased lateral ventricular volumes by alcohol injection

To the best of our knowledge, this is the first study that showed an acute increase in the lateral ventricular volumes by moderate‐dose alcohol injection in healthy subjects, which is in line with previous animal studies.^22^, ^23^ Mechanisms of acute ventricular expansion are poorly understood but may be influenced by CSF, vascular, and brain tissue properties.^24^ Alcohol appears to affect water movement by acutely effecting a hyperosmotic environment leading to shrinkage of brain cells.^25^, ^26^ Zahr et al.^23^ reported the association between increased ventricle volumes and decreased magnetic resonance spectroscopy‐derived tissue water T2 after 4 days of alcohol injection in rats. Since tissue water T2 reflects altered tissue water content, the ventricles were suggested to enlarge in order to compensate for water movement out of tissue in the presence of high blood alcohol levels. However, a previous human study did not find a difference in cerebral water content after alcohol consumption.^9^ This inconsistency can be considered due to the alcohol dosage since animal studies administered about 5 g/kg of alcohol, which is 3–6 times higher than the dose in human study (0.8–1.6 g/kg). According to this hypothesis, enlargement of ventricle volumes may be associated with GM volume reduction. However, increased volumes in the lateral ventricle did not significantly correlate with reduction of total GM volume in our study although both ventricular expansion and GM reduction were found in alcohol‐injected subjects.

Another possibility is that brain ventricles enlarge due to the alcohol effect on CSF flow, turnover, or production. An animal study by Lundgaard et al.^27^ showed that low doses of alcohol (0.5 g/kg) acutely increased CSF tracer influx and clearance, and this effect persisted after 30 days of chronic exposure compared to normal saline, suggesting that glymphatic clearance capability is increased by low doses of alcohol. Conversely, intermediate (1.5 g/kg) and very high dose (4 g/kg) of alcohol exposure decreased glymphatic function after both acute and chronic exposure.^27^ Moreover, a previous human study suggested that acute alcohol administration (1.6 g/kg) produces biphasic increase in the CSF pressure.^28^ CSF flow dynamics in the human brain are not well understood; however, CSF pulsation could be induced by variations in cerebral blood volume resulting from changes in arterial pressure.^29^, ^30^, ^31^ Additionally, reductions in cerebral blood flow, indicated by widespread decreases in fMRI signals, have been associated with transient CSF inflow to the brain.^32^ Recent research suggests that mechanisms dependent on the action of vascular smooth muscle cells, such as neuronal activity, changes in intravascular CO_2_, and autonomic activation from the brainstem, may also induce CSF pulsation.^32^, ^33^ Therefore, cardiovascular and cerebrovascular activities could be crucial determinants of CSF flow.

Numerous studies have demonstrated that alcohol significantly affects the cardiovascular system.^34^ Acute administration of alcohol is known to increase heart rate and reduce average stroke volume, while it generally does not significantly alter systolic and mean blood pressure.^35^, ^36^, ^37^, ^38^ Regarding cerebral hemodynamics, low‐to‐moderate doses of alcohol are found to dilate cerebral arterioles^39^ and enhance cerebral blood flow,^40^, ^41^ whereas high doses have been associated with vasoconstriction.^42^, ^43^ Consequently, alterations in CSF flow, potentially resulting from alcohol's impact on the vascular system, might contribute to ventricular enlargement. However, the precise relationships among changes in the vascular system, CSF flow, and ventricular volumes remain unclear, necessitating further studies to elucidate the mechanisms through which alcohol consumption leads to increased ventricular volumes.

### 4.2 Decreased volumes in the precentral cortex and caudal middle frontal cortex by alcohol injection

We found decreased cortical volumes in the total GM, precentral, caudal middle frontal, and superior frontal cortex after alcohol administration. On the other hand, the only one study thus far by Furtmann et al.^9^ examined the acute brain volume change after heavy alcohol administration and showed no volumetric changes in GM or white matter. These inconsistent results may be observed because Furtmann et al. analyzed only specific regions such as occipital and frontal lobes, thalamus, and pons while we comprehensively examined whole brain regions. For chronic alcohol exposure, accumulating evidence suggested widespread shrinkage of both cortical gray and white matter.^44^ A recent meta‐analysis reported that patients with AUD had GM volume loss in the cingulate and medial frontal cortex, paracentral lobes, left post‐ and precentral cortex, left anterior and right posterior insula, and left superior frontal cortex.^6^ Our findings of cortical volume reductions were observed in the region consistent with this meta‐analysis. However, while the effect of chronic alcohol exposure is associated with brain atrophy, acute alcohol injection possibly affects GM volumes in different ways, considering volume reductions were rapidly recovered by 90 min after injection. Those mechanisms of brain structural alteration via acute alcohol injection and relationship with chronic atrophy are unknown. One possibility of volumetric reductions in cortex is water movement out of tissue and shrinkage of brain cells, as discussed above,^25^, ^26^ but the relationship between tissue water content and volumetric changes caused by acute alcohol administration remains unclear, which warrants further study.

### 4.3 Limitations

First, a total sample size of 18 may be considered small. Second, this study did not include a control group with normal saline injection, which means we cannot rule out the possibility that an increase in body fluids may have influenced brain volumes, particularly ventricular enlargement. According to Zhang et al.,^45^ CSF production is closely linked to the exchange of interstitial fluid, suggesting that an increase in body fluids could indeed enhance CSF production. On the other hand, although no direct studies on humans have examined the impact of isotonic saline on CSF volume, an animal study found no significant changes in CSF flow following saline administration.^46^ These findings highlight the complex interactions between intravenous fluids, CSF production, and brain volume changes. Future studies should include a proper control group to determine whether the observed changes in brain volume are due to alcohol or merely an increase in fluid volume. Third, this study did not measure cerebral blood flow or cardiovascular responses, including heart rate, stroke volume, and blood pressure. Considering the discussed potential associations between alcohol's effects on vascular functions and changes in CSF flow and ventricular volumes, this omission may limit the comprehensiveness of our findings. Fourth, we did not evaluate the alcohol effects on brain volumes beyond 90 min after completement of alcohol injection. Finally, we set the BAC at 0.50 mg/mL, a relatively low level of alcohol.^47^ This may have resulted in small clinical responses measured by VAS to alcohol. Also, the effect on brain structure may vary depending on dosage of alcohol, which warrants further study.

## 5 CONCLUSION

This study revealed the acute effects of alcohol injection on brain volumes in healthy volunteers. Moderate doses of alcohol may increase CSF volumes in the lateral ventricle and decrease volumes in the total GM as well as precentral, caudal middle frontal, and superior frontal cortex, with these changes being reversible. Although alterations in CSF flow and brain tissue water content might be associated with these findings, the mechanisms remain uncertain. We cannot dismiss the possibility that changes in cardiovascular function or overall fluid volume might have influenced the results. Future research should include a suitable control group and measure physiological indicators to further elucidate the underlying mechanisms.

## AUTHOR CONTRIBUTIONS

**Sakiko Tsugawa:** conceptualization (lead), data curation (lead), formal analysis (lead), software (lead), methodology (lead), visualization (lead), and writing—original draft preparation (lead). **Fumihiko Ueno:** data curation (equal), investigation (equal), methodology (equal), project administration (supporting), and writing—review and editing (equal). **Mutsuki Sakuma:** investigation (lead), writing—review and editing (supporting). **Hideaki Tani:** supervision (supporting), and writing—review and editing (equal). **Ryo Ochi:** data curation (supporting), software (supporting), and writing—review and editing (equal). **Ariel Graff‐Guerrero:** supervision (supporting), and writing—review and editing (equal). **Yoshihiro Noda:** supervision (supporting), and writing—review and editing (equal). **Hiroyuki Uchida:** conceptualization (equal), methodology (equal), project administration (equal), supervision (equal), and writing—review and editing (equal). **Masaru Mimura:** supervision (supporting), and writing—review and editing (equal). **Shunji Oshima:** conceptualization (lead), funding acquisition (lead), investigation (supporting), methodology (lead), project administration (lead), resources (lead), supervision (equal), and validation (equal). **Sachio Matsushita:** conceptualization (lead), funding acquisition (lead), investigation (lead), methodology (lead), project administration (lead), resources (lead), supervision (equal), and validation (equal). **Shinichiro Nakajima:** conceptualization (equal), methodology (equal), project administration (lead), supervision (lead), validation (lead), writing—review and editing (lead).

## FUNDING INFORMATION

This research was funded by Asahi Quality and Innovations, Ltd.

## CONFLICT OF INTEREST STATEMENT

Author Hiroyuki Uchida and Shinichiro Nakajima are Editorial Board members of Neuropsychopharmacology Reports and co‐authors of this article. To minimize bias, they were excluded from all editorial decision‐making related to the acceptance of this article for publication. Sakiko Tsugawa has received a fellowship from Nakatani Foundation. Fumihiko Ueno has received fellowship grants from Discovery Fund, Nakatani Foundation, and the Canadian Institutes of Health Research (CIHR); manuscript fees from Dainippon Sumitomo Pharma; and consultant fees from VeraSci, and Uchiyama Underwriting within the past 3 years. Hideaki Tani has received fellowship from the Japanese Society of Clinical Neuropsychopharmacology and the Canadian Institutes of Health Research, a research grant from Eli Lilly, and manuscript fees from Dainippon Sumitomo Pharma, Otsuka Pharmaceutical, Wiley Japan, and Yoshitomi Yakuhin. Yoshihiro Noda has received grants from the Japan Society for the Promotion of Science (JSPS), Japan Agency for Medical Research and Development (AMED), TEIJIN PHARMA LIMITED, Inter Reha Co., Ltd., Japan Health Foundation, Meiji Yasuda Mental Health Foundation, Mitsui Life Social Welfare Foundation, Takeda Science Foundation, SENSHIN Medical Research Foundation, Health Science Center Foundation, Mochida Memorial Foundation for Medical and Pharmaceutical Research, Taiju Life Social Welfare Foundation, and Daiichi Sankyo Scholarship Donation Program. Yoshihiro Noda has received speaker's honoraria from Dainippon Sumitomo Pharma, MOCHIDA PHARMACEUTICAL CO., LTD., Yoshitomi Yakuhin Corporation, Qol Co., Ltd., TEIJIN PHARMA LIMITED, and Takeda Pharmaceutical Company Limited within the past 5 years. Yoshihiro Noda also receives equipment‐in‐kind support for an investigator‐initiated study from Magventure Inc., Inter Reha Co., Ltd., Brainbox Ltd., and Miyuki Giken Co., Ltd. Hiroyuki Uchida has received grants from Eisai, Otsuka Pharmaceutical, Dainippon‐Sumitomo Pharma, Daiichi Sankyo Company, and Mochida Pharmaceutical; speaker's honoraria from Otsuka Pharmaceutical, Dainippon‐Sumitomo Pharma, Eisai, Janssen Pharmaceuticals, Lundbeck Japan, and Meiji‐Seika Pharma; and advisory panel payments from Dainippon‐Sumitomo Pharma and Lundbeck Japan within the past 3 years. Masaru Mimura has received research support from JSPS and grants or speaker's honoraria from Daiichi Sankyo, Dainippon‐Sumitomo Pharma, Eisai, Eli Lilly, Fuji Film RI Pharma, Janssen Pharmaceutical, Mochida Pharmaceutical, MSD, Nippon Chemipher, Novartis Pharma, Ono Yakuhin, Otsuka Pharmaceutical, Pfizer, Takeda Yakuhin, Tsumura, and Yoshitomi Yakuhin within the past 3 years. Sachio Matsushita has received a research grant from Asahi Breweries and has received speaker's honoraria from EA Pharma, Ono Yakuhin, Otsuka Pharmaceutical, and Yoshitomi Yakuhin within the past 3 years. Shinichiro Nakajima has received grants from JSPS, AMED, Japan Research Foundation for Clinical Pharmacology, Naito Foundation, Takeda Science Foundation, Uehara Memorial Foundation, and Daiichi Sankyo Scholarship Donation Program within the past 3 years. Shinichiro Nakajima has also received research support, manuscript fees or speaker's honoraria from Dainippon Sumitomo Pharma, Meiji‐Seika Pharma, Otsuka Pharmaceutical, Shionogi, and Yoshitomi Yakuhin within the past 3 years. Other authors declare no conflict of interest.

## ETHICAL APPROVAL

Approval of the Research Protocol by an Institutional Reviewer Board: The study procedures were conducted in accordance with the guidelines set forth in the Declaration of Helsinki. Ethical clearance was obtained from the review board of the National Hospital Organization Kurihama Medical and Addiction Center, Yokosuka, Japan (Approval ID: 320‐2) and Keio University School of Medicine, Tokyo, Japan (Approval ID: 20180112).

Informed Consent: Written informed consent was obtained from all participants after providing a detailed explanation of the study's purpose and procedures. The participant's personal information was kept anonymous.

Registry and the Registration No. of the Study: The study protocol was registered with the University hospital Medical Information Network Clinical Trials Registry (ID: UMIN000032753).

Animal Studies: N/A.

## Supporting information


Tables S1–S2:

Data S1:

## Data Availability

The data that support the findings of this study are available in a Data S1.
