# Supplementary material for: Effects of short‐term exposure to moderate amounts of alcohol on brain volume
Source: Neuropsychopharmacol Rep. 2024 Dec 12;45(1):e12500. doi: 10.1002/npr2.12500 (PMC11772103; doi:10.1002/npr2.12500)
Supplement: Supplementary file 1 — Tables S1–S2: [file NPR2-45-e12500-s002.docx]

**Supplementary Table 1.** Contents of clinical assessments with the visual analogue scale

| Clinical symptom | Category |
| --- | --- |
| Up^a^ | Stimulant |
| Stimulated^a^ |  |
| Energized^a^ |  |
| Excited^a^ |  |
| Talkative^a^ |  |
| Elated^a^ |  |
| Vigorous^a^ |  |
| Difficulty concentrating^a,b^ | Sedative |
| Slow thoughts^a^ |  |
| Down^a^ |  |
| Heavy head^a^ |  |
| Inactive^a^ |  |
| Sedated^a,b^ |  |
| Relaxed^b^ |  |
| Impaired writing^b^ |  |
| Numb all over^b^ |  |
| Heavy^b^ |  |
| Face numb^b^ |  |
| Tired^a^ |  |
| Warm^b^ | Physical |
| Heart beat changing^b^ |  |
| Face flush^b^ |  |
| Head spinning^b^ |  |
| Ringing, buzzing^b^ |  |
| Sick |  |
| Burning in stomach^b^ |  |
| Headache |  |
| Diarrhea |  |
| Nauseous^b^ |  |
| Craving | Craving |

a. The items were quoted from the Biphasic Alcohol Effects Scale [(Martin et al., 1993)](https://paperpile.com/c/Uicamo/usSrF).

b. The items were quoted from the Bodily Sensation Scale [(Maisto et al., 1980)](https://paperpile.com/c/Uicamo/4rZVI)

**Supplementary Table 2.** Changes in the VAS scores through 180 min

| VAS scores |  |  |  |  |  |  |
| --- | --- | --- | --- | --- | --- | --- |
| Timepoint, min | 0 | | 90 | | 180 | |
| Stimulant, mean (SD) cm | 3.3 (1.6) | | 3.3 (1.6) | | 3.3 (1.6) | |
| Sedative, mean (SD) cm | 2.4 (1.4) | | 4.9 (1.7) | | 3.8 (1.5) | |
| Physical, mean (SD) cm | 1.0 (1.1) | | 3.1 (1.4) | | 2.2 (1.2) | |
| Craving, mean (SD) cm | 0.7 (0.6) | | 1.3 (1.6) | | 0.7 (0.5) | |

**SUPPLEMENTARY REFERENCES**

[Maisto SA, Connors GJ, Tucker JA, McCollam JB, Adesso VJ (1980) Validation of the Sensation Scale, a measure of subjective physiological responses to alcohol. Behav Res Ther 18:37–43.](http://paperpile.com/b/Uicamo/4rZVI)

[Martin CS, Earleywine M, Musty RE, Perrine MW, Swift RM (1993) Development and validation of the Biphasic Alcohol Effects Scale. Alcohol Clin Exp Res 17:140–146.](http://paperpile.com/b/Uicamo/usSrF)
